# Editorial: The immunobiology of the skin in response to the environment

**DOI:** 10.3389/fimmu.2023.1220335

**Published:** 2023-05-22

**Authors:** Elena Donetti, Michele Sommariva, Andrea Chiricozzi, Francesca Prignano

**Affiliations:** ^1^ Department of Biomedical Sciences for Health, Università degli Studi di Milano, Milan, Italy; ^2^ Dermatologia, Dipartimento Universitario di Medicina e Chirurgia Traslazionale, Università Cattolica del Sacro Cuore, Rome, Italy; ^3^ Dermatologia, Dipartimento Scienze Mediche e Chirurgiche, Fondazione Policlinico Universitario A. Gemelli IRCCS, Rome, Italy; ^4^ Department of Health Sciences, Section of Dermatology, University of Florence, Florence, Italy

**Keywords:** psoriasis, atopic dermatitis, wound healing, human skin, antioxidants

Due to its strategic anatomical location, the human skin faces at the same time the body’s internal organs and the environment, thus playing a crucial role as a powerful biological barrier. For this reason, it is endowed with equipment to rapidly respond to a plethora of physiopathological events, among which are several types of mechanical injuries, wound healing, and different inflammatory diseases, such as atopic dermatitis and psoriasis (1).

In this Research Topic, the multifaceted skin response in different experimental and clinical conditions was reported in two *Original Research* and two *Review*.

At present, the lack of efficacious cures for psoriasis and atopic dermatitis represents a clinical unmet need and the discovery of novel therapeutic strategies gained a lot of attention in the last years as witnessed by the studies included in this Research Topic reporting the beneficial effect of natural compounds in experimental models mimicking these dermatological diseases.

Roy et al. investigated the effect of fisetin, a natural dietary polyphenol present in colored fruits and vegetables, possessing anti-oxidant and anti-inflammatory properties. Using a cellular model of primary normal human epidermal keratinocytes exposed to a proinflammatory microenvironment, mediated by IL17A/TNFα, that recapitulates the situation occurring in psoriatic skin, the Authors discovered that fisetin had a profound impact on keratinocyte transcriptome by altering the mTOR signaling pathway, reported to be involved in psoriasis onset by promoting psoriatic lesions and keratinocyte hyperplasia (2). Subsequent experiments showed that fisetin acts by down-modulating Akt/mTOR signaling pathway and, at the same time, by stimulating autophagy thus leading to a decrease in the proinflammatory phenotype of keratinocytes. The biological activity of fisetin is not only restricted to keratinocytes but also acts on immune cells. In particular, this molecule has been observed to reduce the production and secretion of IL17A by activated CD4^+^ lymphocytes. Moreover, fisetin topical treatment improved skin lesions in a mouse model of imiquimod-induced psoriasis. Accordingly, immunohistochemical analysis revealed that the anti-inflammatory property of this compound relies on the ability to decrease Akt/mTOR signaling, corroborating *in vitro* findings. This effect was paralleled by a reduction of immune cell infiltration and the release of pro-inflammatory mediators in the skin. Collectively, the work by Roy et al. provides interesting insights into the use of fisetin for the treatment of patients affected by psoriasis and possibly other inflammatory skin diseases.

The paper by Singh et al. reported the beneficial effect of dietary grape powder in an *in vivo* murine model mimicking atopic dermatitis, thus suggesting that grape antioxidants in their natural combination can induce synergistic interactions among the many different components present in grapes. This study strongly supports the use of a common nutrient as an alternative or supplemental strategy in the successful management of AD.

Wound healing and scar formation represent physiological processes that are closely linked as injured skin generally heals through the deposition of fibrotic scar tissue. The ideal healing process leads to skin regeneration recovering pre-injury skin features, with restored ultrastructure and mechanical properties. However, injured skin may heal through the formation of fibrotic scar tissue, sometimes represented by hypertrophic scarring, that could be associated with an unaesthetic appearance in visible areas with possible psychological distress for patients. Wound healing is an important biologic process with the final purpose to restore well-differentiated and functional tissue. It is characterized by many steps, during which cells, pro-inflammatory cytokines, and soluble factors play a very peculiar and specific role that has to be perfectly balanced in order to achieve good results. Wound healing is normally divided into phases to better face the main cellular processes which characterize every single phase. The main steps are inflammation, proliferation, and remodeling: they can overlap and be interdependent but it is a cultural way to address the specific molecular/cellular events which characterize these processes.

Yin et al. proposed a detailed overview of the wound healing process, describing the role of both immune and tissue cells involved in the different stages of the process. Nevertheless, they highlighted how an aberrant cell activation or tissue deposition may be responsible for an unfavorable hypertrophic scar formation that usually consists of a fibroproliferative disorder that may arise after deep cutaneous injuries caused by trauma, burns, or surgery. In this scenario, various mechano-transduction signaling pathways, as well as mechanosensitive ion channels, may contribute to wound healing and stretch-induced hypertrophic scar formation. A better understanding of these signaling pathways involved in the transduction of signals induced by mechanical forces may lead to the development of a targeted and more effective therapeutic approach as current therapies do not efficiently attain scar-less healing or reverse fibrosis.

The Review by Wang et al. took into consideration the inflammatory phase of wound healing, with a good characterization of the inflammatory cells in the inflammatory microenvironment. Neutrophils, as the first circulating cells recruited to the microenvironment for bacteria and necrotic tissue removal, are also cells to be removed to prevent secondary necrosis. Macrophages are involved in all stages; it is the surrounding microenvironment that regulates their phenotype and functions. Mast cells, T lymphocytes, platelets, and complement are part of the inflammatory microenvironment. The role of dendritic cells, in particular of Langerhans cells, is highly interesting, as the lack of these antigen-presenting cells and plasmacytoid dendritic cells delays wound healing. Most of the inflammatory cells of the inflammatory microenvironment as non-inflammatory cells (i.e. fibroblasts, keratinocytes) can achieve significant plasticity under the influence of the ECM. A correct remodeling of skin wounds is possible through a fine regulation –not completely elucidated yet- among the ECM, the inflammatory cells, and the non-inflammatory cells.

In conclusion, the scientific goal of this Research Topic is to present new insights describing and dissecting the main cellular and molecular mechanisms able to preserve the morphofunctional skin features, paving the way for the development of novel therapeutic interventions for better management or prevention of skin diseases.

## Author contributions

All authors listed have made a substantial, direct, and intellectual contribution to the work and approved it for publication.
